# Cre recombinase affects calcium dynamics already in young mice

**DOI:** 10.3389/fphar.2025.1558573

**Published:** 2025-03-26

**Authors:** János Levin Liffers, Jan Peter Reinhardt, Matthias Dodo Seidl, Uwe Kirchhefer, Frank Ulrich Müller, Jan Sebastian Schulte

**Affiliations:** Institute of Pharmacology and Toxicology, University of Münster, Münster, Germany

**Keywords:** transgenic mice, cre recombinase, LoxP sites, Myh6-cre, cardiotoxicity, Ca^2+^ dynamics

## Abstract

**Background:**

The Cre/LoxP system is widely used in cardiovascular research to generate mouse models with tissue-specific inactivation of target genes. Studies have reported that expression of Cre recombinase under the αMHC promoter leads to age-dependent cardiotoxicity with ventricular hypertrophy, fibrosis and ventricular dysfunction at 6 months of age. This study explores the impact of Cre expression on intracellular Ca^2+^ dynamics in ventricular myocytes of αMHC-Cre mice as early as 3 months old.

**Methods:**

Mice expressing Cre under the αMHC promoter (CRE) were compared to wild-type (WT) controls. Ventricular cardiomyocytes (VCMs) were isolated by the Langendorff method. Ca^2+^ transients and sarcomere shortening were simultaneously recorded from VCMs. Ventricular and atrial weights were assessed, VCM dimensions analyzed, and protein and mRNA levels of key proteins involved in Ca^2+^ dynamics measured by immunoblot analysis and quantitative real-time RT-PCR.

**Results:**

At 3 months, CRE mice showed no evidence of cardiac hypertrophy. Ventricular or atrial weights and VCM size were not different between CRE and WT mice. The same applied to protein levels of SERCA2a, NCX1, Cav1.2, PLN and its phosphorylated form PLN pThr17. Nevertheless Ca^2+^ dynamics were significantly altered in CRE mice. Under basal conditions resting and peak Ca^2+^ were reduced and Ca^2+^ transient decay was delayed up to 30% in VCMs from CRE vs. WT mice. These differences persisted upon stimulation with 1 µM isoproterenol, whereas Ca^2+^ transient amplitude increased in CRE VCMs. We confirmed a previously reported reduction in dystrophin, potentially explaining the changes in Ca^2+^ dynamics. Despite these changes sarcomere shortening parameters were not different between groups.

**Conclusion:**

As early as 3 months of age, Cre expression in VCMs leads to changes in Ca^2+^ dynamics that do not yet affect sarcomere shortening and cannot be attributed to the regulation of key proteins involved in Ca^2+^ dynamics. Because changes in intracellular Ca^2+^ dynamics can affect gene expression through altered excitation-transcription coupling, researchers should be aware of these subtle changes that precede the prominent phenotype at 6 months of age. Therefore, it is essential to use Cre-positive mice as controls when analyzing knockout models generated by the Cre/LoxP system.

## Introduction

The Cre/LoxP system is widely used to generate mouse models with cell- or tissue-specific inactivation (knockout) of respective target genes. This system exploits the properties of the enzyme Cre recombinase, which recognizes and recombines specific DNA sequences known as loxP sites (McLellan et al., 2017; Sternberg und Hamilton, 1981). When target genes are flanked by two loxP sites oriented in the same direction, Cre recombinase can excise the flanked “floxed” locus, thereby deleting the gene of interest. By mating two different mouse lines - one carrying the gene of interest flanked by loxP sites and the other expressing Cre recombinase under a tissue-specific or inducible promoter - researchers can achieve spatial and temporal control over gene expression. Several consortia, such as the International Knockout Mouse Consortium have engineered cell lines or animals with conditional-ready floxed allels for the majority of protein coding genes in the mouse genome (Skarnes et al., 2011). Mice expressing the Cre recombinase under the control of cell-, tissue-specific or global promoters are also available in large numbers (Kim et al., 2018). The Cre/loxP system therefore provides researchers with a valuable tool to inactivate almost any gene in the mouse genome and to analyze the functional consequences of this deletion. The group of Schneider constructed the first cardiomyocyte-specific Cre-model by linking its expression to the alpha-myosin heavy chain (αMHC, Myh6) promoter (Agah et al., 1997). Other mouse models are now available that express Cre recombinase, e.g., under the control of the cardiac rat troponin T2 promoter (cTntCre) (Jiao et al., 2003), the β-myosin heavy chain promoter (βMHC, Myh7) (Parsons et al., 2004) or the myosin light chain 2v (Myl2, MLC2v) promoter (Chen et al., 1998). Temporal control of Cre expression has been achieved by fusing Cre to the estrogen receptor. Hormone-bound, inactive Cre can be activated by the application of tamoxifen, which induces recombination, and cardiomyocyte-specific variants are available (Sohal et al., 2001; Rashbrook et al., 2022). However, the αMHC-Cre mouse constitutively expressing Cre is the most widely used (Ronda and Roderick, 2024). In recent years, there has been increasing evidence that cardiomyocyte-specific expression of Cre recombinase leads to age-related cardiotoxicity (Buerger et al., 2006; Rehmani et al., 2019; Pugach et al., 2015; Rashbrook et al., 2022; Li et al., 2023), which has been attributed to the presence of pseudo-LoxP sites in the mouse genome (Pugach et al., 2015; Burashnikov and Antzelevitch, 2010; Thyagarajan et al., 2000). In 6-month-old αMHC-Cre mice authors observed reduced ventricular function (Pugach et al., 2015; Rehmani et al., 2019) dilated cardiomyopathy (Rehmani et al., 2019), fibrosis (Li et al., 2023; Pugach et al., 2015; Garbern et al., 2019), and ventricular hypertrophy (Pugach et al., 2015; Li et al., 2023). This was preceded by increased expression of Anp (atrial natriuretic peptide) and Bnp (brain natriuretic peptide) already in 3-month-old αMHC-Cre mice, in which no significant ventricular hypertrophy or dilatation had yet been observed (Pugach et al., 2015; Rehmani et al., 2019; Garbern et al., 2019). However, Gillet et al. reported a reduction in the amplitude of the L-type Ca^2+^ current, which is critical for regular excitation-contraction-coupling, in ventricular cardiomyocytes isolated from 3-month-old αMHC-Cre mice compared to wild-type, before significant signs of cardiotoxicity become apparent (Gillet et al., 2019). We therefore speculated that Cre expression might affect excitation-contraction-coupling in ventricular cardiomyocytes at an age when overt manifestations of cardiotoxicity are not yet visible. Here, we report that Cre expression affects the intracellular Ca^2+^ dynamics in ventricular cardiomyocytes from 3-month-old αMHC-Cre mice, without obvious regulation of key proteins of the Ca^2+^ homeostasis, while myocyte shortening is still unaffected.

## Methods and materials

### Animals

Wild-type FVB/N mice (WT, Strain: FVB/NHanHsd) were bred with mice that exhibited heterozygous expression of Cre recombinase under the control of the cardiomyocyte-specific α-myosin heavy chain promoter (αMHC^Cre+/−^mice, CRE) (Agah et al., 1997) backcrossed to the same FVB/N strain. WT and CRE littermates of both sexes were analyzed at an age of 12–14 weeks. The applied experimental methods confirmed to the instructions of Directive 2010/63/EU of the European Parliament on the protection of animals used for scientific purposes and were approved by the local authorities (Landesamt für Natur, Umwelt und Verbraucherschutz NRW; permission number: 53.5.32.7.1/MS-07842 and 81-02.05.50.20.012).

### Study design

The investigators were blinded to the genotypes of the mice used for myocyte isolation. Following the completion of data analysis, the genotypes were revealed to perform the respective statistics. Hearts were collected for determination of heart weights, immunoblotting and mRNA quantification in an unblinded, explorative approach.

### Isolation of ventricular cardiomyocytes

The methodology for isolating ventricular cardiomyocytes (VCMs) have been described before (Schulte et al., 2012; Bögeholz et al., 2018). Briefly, mice were sacrificed, hearts immediately removed, prepared and connected to a modified Langendorff apparatus to be perfused with collagenase/protease solution at 37°C at a constant flow rate of 2.5 mL/min. A collagenase activity of 190 U/mL and a protease activity of 0.3 U/mL were used (Collagenase Type II, 265 U/mg, Worthington; Protease Type XIV, 5.4 U/mg, Sigma). After sufficient perfusion time the heart tissue was carefully dissected with forceps and VCMs were released. For functional analysis Ca^2+^ was stepwise increased to a final concentration of 1 mM and VCMs stored at room temperature.

### Determination of heart weights and myocyte dimensions

To determine relative heart weights, WT and CRE mice were sacrificed and first weighed as a whole. The hearts were then removed and separated into atria and ventricles, which were weighed separately using an analytical balance. To determine VCM’s dimensions, isolated VCMs were pipetted into an organ bath and transferred to the stage of an automatized microscope (Nikon Ti-E, Tokyo, Japan). Images (5 × 2 neighboring fields of view) were taken at ×20 magnification and merged. These overview images were captured at disparate positions in the organ bath. The cell length and cell width of 50 neighboring VCMs per isolation were then analyzed using NIS-elements AR (NIS-Elements Advanced Research, Nikon, Tokyo, Japan).

### Measurement of intracellular Ca^2+^ and sarcomere shortening

Intracellular Ca^2+^ and sarcomere shortening were analyzed using a Myocyte Calcium and Contractility System from Ionoptix (Ionoptix, Milton, MA, United States) as described before (Schulte et al., 2016). Isolated VCMs were loaded with the Ca^2+^ indicator Indo-1/AM (9 µM) (Molecular Probes^®^, Thermo Fisher Scientific, Waltham, MA, United States) for 10 min at room temperature. After incubation VCMs were placed in a pacing chamber on the stage of an inverted microscope (Eclipse Ti-S, Nikon, Tokyo, Japan) and perfused with Tyrode’s solution (140 mM NaCl, 5.8 mM KCl, 0.5 mM KH_2_PO_4_, 0.4 mM Na_2_HPO_4_ x2H_2_O, 0.9 mM MgSO_4_ x7H_2_O, 10 mM HEPES, 10 mM glucose, 2 mM CaCl_2_, pH 7.4 adjusted with NaOH) and field stimulated at 0.5 Hz (Myopacer, Ionoptix, Milton, MA, United States) at room temperature. Ca^2+^ was determined by measuring the Indo-1 ratio (405/495 nm) at 340 nm excitation using an UVICO flash lamp (Rapp OptoElectronic GmbH, Wedel, Germany) equipped with appropriate filters. Sarcomere shortening was simultaneously recorded with a digital camera (Myocam-S, Ionoptix, Milton, MA, United States). Ca^2+^ transients and sarcomere shortening were recorded from at least 10 cardiomyocytes first under basal conditions and then during perfusion with 10^−6^ M isoproterenol (ISO). Data was analyzed using Ionwizard software (Ionoptix, Milton, MA, United States).

### Immunoblotting and quantitative real-time RT-PCR

Ventricular homogenates were prepared from explanted, shock frozen hearts using NaHCO_3_/SDS buffer. Protein content was determined according to the Lowry method, with bovine serum albumin (BSA) used as the standard. Individual samples were electrophoretically separated on 4%–15% tris-glycine gradient gels (Criterion™ TGX™ precast gels, Bio-Rad, California, United States) and subsequently transferred to nitrocellulose membranes. The concentration of each sample was 30–60 μg. Ponceau-S staining was performed immediately after the transfer. For blocking, membranes were treated with 5% dry milk in TBS-T for 1 h at room temperature. When utilizing phosphorylation-specific antibodies, blocking was conducted with 5% BSA. Target proteins were probed using the following primary antibodies: Cav1.2 (Alomone ACC-003, 1:300), SERCA2a (abcam ab150435, 1:100,000), NCX1 (Swant R3F1, 1:1,000), total PLN (Badrilla A010-14, 1:2,000), PLN pThr17 (Badrilla A010-13AP, 1:2,000), PLN pS16 (Badrilla A010-12AP, 1:2,000), Dystrophin (Proteintech 83609-5-RR, 1:20,000). An anti-mouse IgG-HRP antibody (GE-Healthcare NA931 V) was employed as the secondary antibody for NCX, while an anti-rabbit IgG-HRP antibody (GE-Healthcare NA934 V) was utilized for all other proteins. Protein bands were visualized using the SERVALight Helios substrat (SERVA Electrophoresis GmbH, Heidelberg, Germany). Images were acquired using the ChemiDoc™ MP imaging system (Bio-Rad Laboratories GmbH) and analyzed using Image Lab™ (Bio-Rad Laboratories GmbH) and TotalLab™ Quant (TotalLab Ltd., United Kingdom) software. Band intensities of target proteins were normalized to the corresponding summed lane intensity of Ponceau S staining for total protein normalization (Sander et al., 2019). Relative PLN phosphorylation was calculated as the ratio normalized phosphoprotein/normalized total PLN, with the WT ratio set to 1.

For mRNA quantification, 10 snap-frozen ventricular samples were first pulverized and 20–25 mg per sample was taken into 750 µL TRIzol™ (Thermo Fisher Scientific). RNA was purified using the Direct-zol™ RNA MiniPrep Kit (Zymo Research), without DNAse digestion according to the manufacturer’s instructions. 1 μg of RNA per sample was reverse transcribed to cDNA using the LunaScript RT SuperMix Kit (New England Biolabs GmbH, Germany). Quantitative real-time PCR (qRT-PCR) was performed using a Light-Cycler 480 II system (Roche Applied Science). Reactions were set up in a 96-well plate containing a mixture of 1 μL isolated cDNA, 0.5 μL of each primer (10 p.m., each), 10 μL Luna^®^ Universal qPCR Master Mix (New England Biolabs GmbH) and 8 μL H_2_O. For amplification, the samples were incubated at 95°C for 5 min, followed by 45 cycles of 95°C for 10 s, 60°C for 15 s and 72°C for 20 s. To determine the efficiency of the reaction, calibration curves were generated with different amounts of control cDNA. Crossing points were determined by the second derivative method using LightCycler 480 software version 1.5.1.62. After amplification, a melting curve analysis was performed to check the specificity of the reaction. Relative quantification was performed by calculating relative expression ratios using the ΔΔC_T_ method, taking into account the efficiency of the reaction using the relative expression software tool (REST, version 2.013) (Vandesompele et al., 2002). The statistical random analysis was performed with 5,000 iterations. Hprt1 and Ywhaz were used as reference genes. The following primer pairs were used for amplification: *Hprt* F-5′- ATG​AGC​GCA​AGT​TGA​ATC​TG, *Hprt* R-5′-GGACGCAGCAACTGACATT, *Ywhaz* F-5′-TTGAGCAGAAGACGGAAGGT, *Ywhaz* R-5′- GAA​GCA​TTG​GGG​ATC​AAG​AA, *Atp2a2* F-5′- CTG​TGG​AGA​CCC​TTG​GTT​GT, *Atp2a2* R-5′- CAG​AGC​ACA​GAT​GGT​GGC​TA, *Cacna1c* F-5′- GCT​CTC​TTC​ACC​GTC​TCC​AC, *Cacna1c* R-5′- GAC​GAA​ACC​CAC​GAA​GAT​GT, *Scn5a* F-5′- TAC​CGC​ATA​GTG​GAG​CAC​AG, *Scn5a* R-5′- ATC​TCG​GCA​AAG​CCT​AAG​GT, *Slc8a1* F-5′- CAT​TTG​AGG​AAC​CCG​TGA​CT, *Slc8a1* R-5′- GAA​TTC​GAG​CTC​TCC​ACA​GG, *Pln* F-5′- TTG​GAA​ACA​GGT​TTG​CAT​GA, *Pln* R-5′- TCA​CGT​TTC​TCT​CAG​CAT​GG, *Ryr2* F-5′- TAA​TGG​TCT​CCT​TGC​AGC​CA, *Ryr2* R-5′- TTC​GGA​TGG​CTT​CTC​CCT​TT, *Dmd* F-5′- TCC​TCT​CCT​TCC​ACC​TCT​C, *Dmd* R-5′- TCT​AAC​CCT​GTG​CTT​GTG​TC, *Casq2* F-5′ – CTT​TGC​GGA​GAA​GAG​TGA​CC, *Casq2* R-5′ – ACA​TTC​ACC​ACC​CCA​ATC​TG, *Trdn* F-5′ – AAG​AGC​CCT​TGT​CAT​ACC​CC, *Trdn* R-5′ – GAC​AGA​CCT​CTT​CAG​CAC​CT, *Canx* F-5′ – CTT​TGC​CAG​TGT​TCC​TTG​TG, *Canx* R-5′ – CTTCCT CTTCATCCCTCTTGTTC, *Kcnj2* F-5′ – GGG​AAT​TCT​CAC​TTG​CTT​CG, *Kcnj2* R-5′ – AGA​GAT​GGA​TGC​TTC​CGA​GA.

### Data presentation and statistical analysis

Numerical data are presented as mean ± standard deviation. In the figures, data are presented as box plots. Boxes represent the data between the 25th and 75th percentiles, whiskers represent data between the 10th and 90th percentiles, horizontal lines indicate the median value, while the mean is indicated by the small square. The raw or aggregated data points appear as an overlay. For the analysis of heart weight, protein and mRNA levels each data point corresponds to a single independent sample. For the analysis of VCM dimensions each data point corresponds to the median of 50 analyzed VCMs, and for the analysis of Ca^2+^ transients and sarcomere shortening, each data point corresponds to the median of 10 measured VCMs. These aggregated data were also used for statistical analysis. To compare two independent groups, the unpaired two-tailed Student’s t-test or Mann-Whitney rank sum test was used, depending on the presence of normal distribution and equal variances. P values <0.05 were considered statistically significant.

## Results

### Cre-expressing mice show no hypertrophy at 3 months of age

**FIGURE 1:**
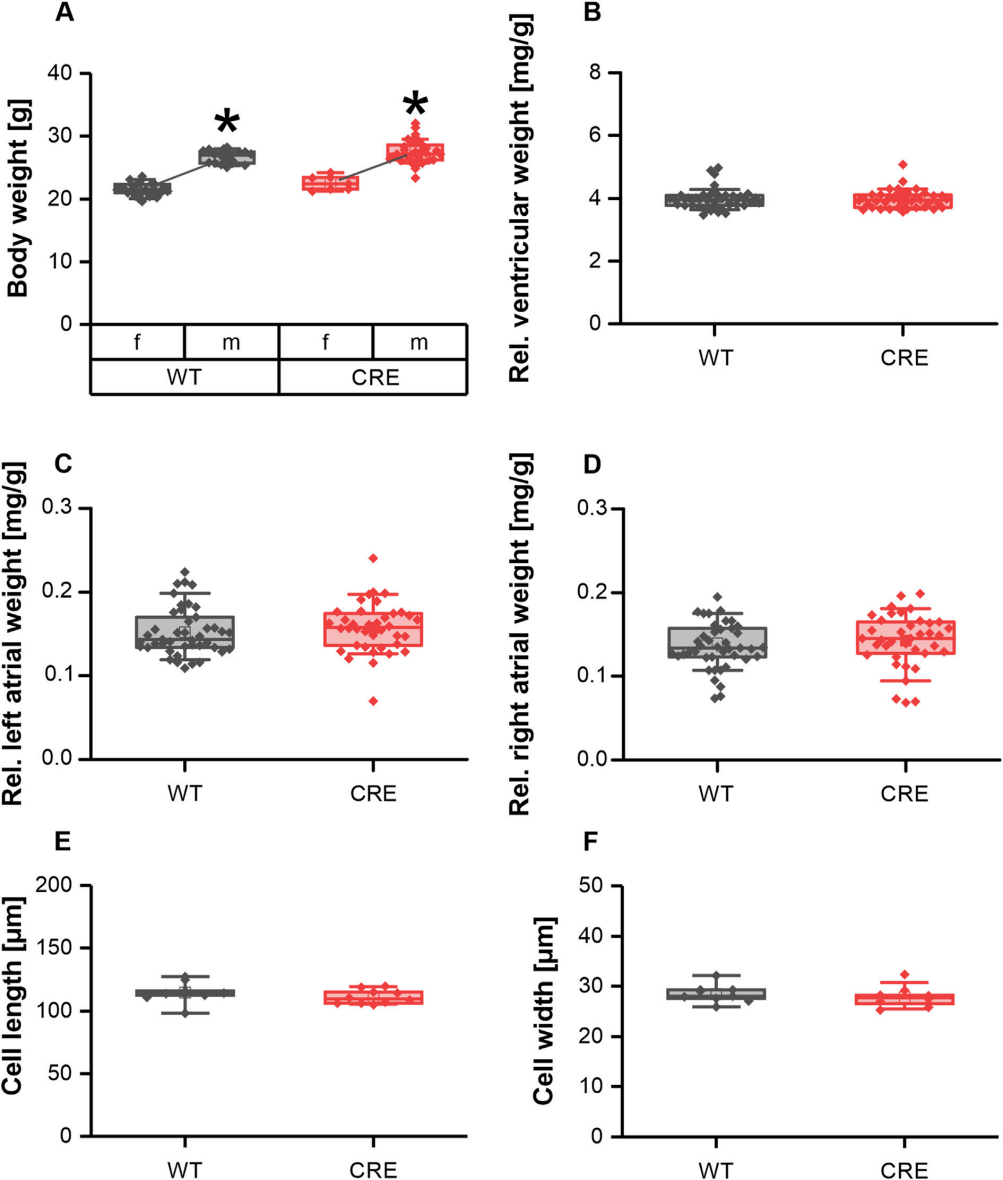
No signs of hypertrophy in 3-month-old mice with Cre-expression. Body weight **(A)** differed between sexes but was not different between genotypes [number of mice, male (m)/female (f), WT: n = 18/25; CRE: n = 31/7; *p < 0.05 m vs. female f)]. Ventricular weight **(B)** left atrial weight **(C)** and right atrial weight **(D)** relative to body weight were not affected by Cre expression in 3-month-old mice. At the same time VCM length **(E)** and width **(F)** were not different between groups (number of Isolations, m/f, WT: n = 5/4; CRE: n = 5/5; each data point represents the median of 50 analyzed VCMs per isolation).

It has been reported that expression of Cre recombinase under control of the αMHC promotor leads to cardiac hypertrophy in mice as early as 6 months of age (Li et al., 2023). Here we investigated parameters of cardiac hypertrophy at a much younger age. Body weight was not different between CRE and WT mice at 3 months of age, regardless of sex (Figure 1A). The relative ventricular and atrial weights of explanted hearts normalized to body weight were also unchanged between groups (Figures 1B–D), indicating no macroscopic evidence of cardiac hypertrophy. In line with this, cell length (Figure 1E) and cell width (Figure 1F) of isolated ventricular cardiomyocytes (VCMs) were unchanged between groups. Consequently, at 3 months of age, there is no evidence of ventricular hypertrophy in mice expressing αMHC-Cre, considering our *in vitro* data.

### Cre expression leads to changes in Ca^2+^ dynamics under basal conditions before the manifestation of hypertrophy

**FIGURE 2:**
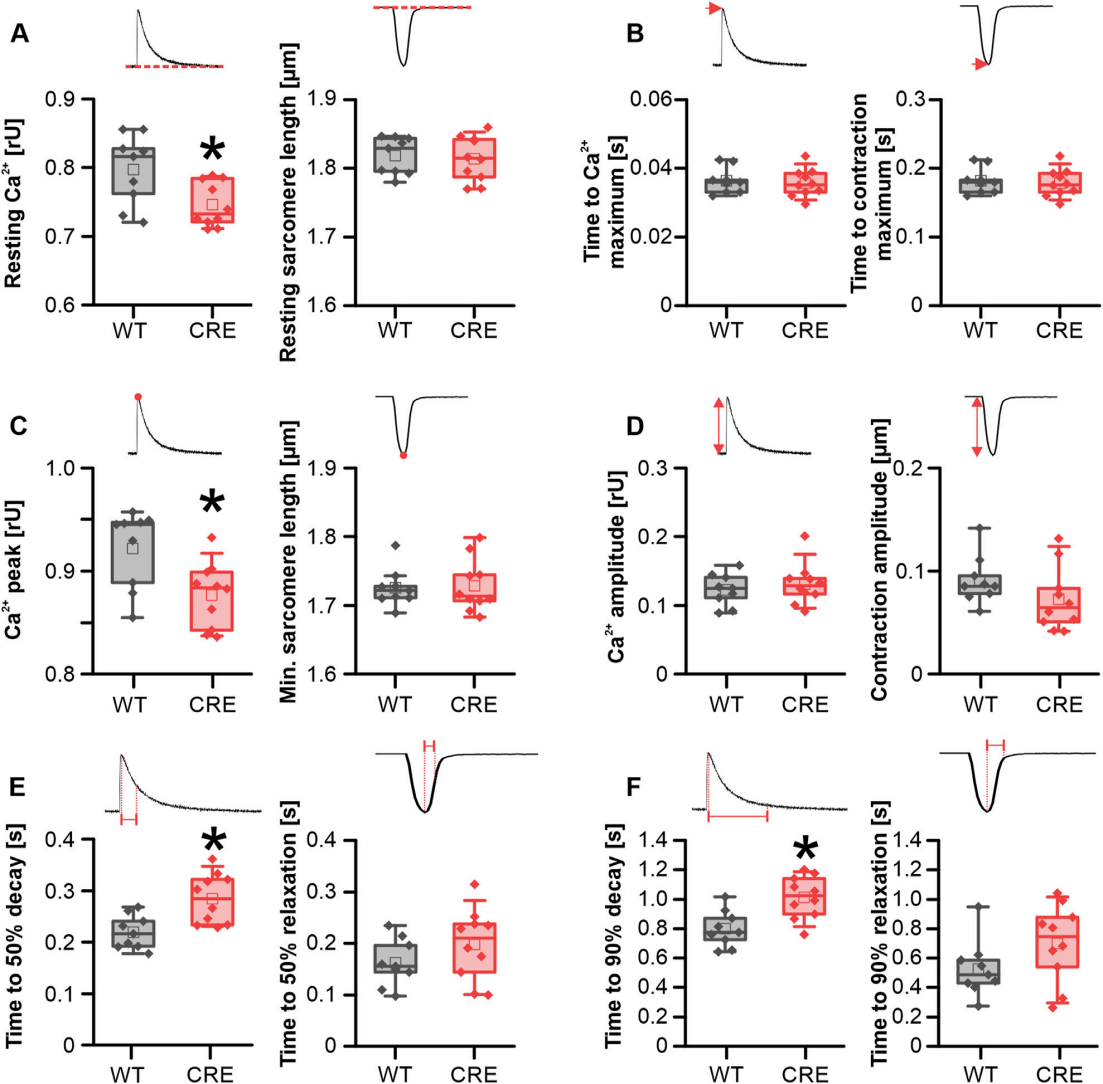
Ca^2+^ dynamics is altered in CRE-mice under basal conditions. Each panel shows the indicated parameter for Ca^2+^ transients (left) and sarcomere shortening (right) under basal conditions in VCMs isolated from CRE and WT mice. Resting Ca^2+^ **(A)** and peak Ca^2+^ levels **(C)** were reduced in VCMs from CRE vs. WT mice. Given that both parameters exhibited a similar reduction, the Ca^2+^ transient amplitude **(D)** was not different between groups. Moreover, cytosolic Ca^2+^ removal was delayed in the CRE group as evidenced by an increased time to 50% **(E)** and 90% **(F)** decay of the Ca^2+^ transient in VCMs from CRE vs. WT. mice. Ca^2+^ release speed was not affected by Cre expression since the time to maximum of the Ca^2+^ transient **(B)** was not different between groups. All shortening parameters [**(A–F)**, right] were not affected by Cre expression (Number of isolations, m/f, WT: n = 5/4; CRE: n = 5/5; each datapoint represents the median of 10 analyzed VCMs per isolation; *p < 0.05 vs. WT).

Hypertrophic remodeling of cardiomyocytes is often accompanied by changes in Ca^2+^ homeostasis. We therefore investigated whether changes in Ca^2+^ dynamics already occur before the macroscopic appearance of hypertrophy. To analyze this, intracellular Ca^2+^ was assessed using the Ca^2+^-sensitive dye Indo-1 (ratio 405/495 nm), while the sarcomere shortening was recorded using a CCD camera simultaneously. The resting Ca^2+^ level was reduced in VCMs from CRE versus WT mice by 6.4% (Figure 2A). Because peak Ca^2+^ was also reduced by almost the same amount in the CRE group (Figure 2C), the Ca^2+^ transient amplitude was not altered between groups (Figure 2D). At the same time, the speed of Ca^2+^ release did not appear to be affected by Cre expression, as evidenced by the lack of alteration in the time to maximum of the Ca^2+^ transient between the two groups (Figure 2B). However, the rate of Ca^2+^ removal from the cytosol was markedly delayed in VCMs with Cre expression. The time required to reach 50% (Figure 2E) and the time required to reach 90% decay of Ca^2+^ (Figure 2F) was increased by 30% and 27%, respectively, in VCMs from CRE versus WT mice. Consistent with a delayed Ca^2+^ removal, the time constant τ of a single exponential decay function fitted to the decay phase of the Ca^2+^ transient and the area under the curve (AUC) of the Ca^2+^ transient during this phase were significantly increased by 32% and 39%, respectively (τ in s, CRE vs. WT, 0.44 ± 0.09 vs. 0.33 ± 0.04, p = 0.003 vs. WT; AUC in relative units*s, CRE vs. WT, 0.055 ± 0.01 vs. 0.039 ± 0.01, p = 0.013 vs. WT; data not shown). At the same time, sarcomere shortening parameters were not significantly different between groups (Figures 2A–E, right panels). However, there was a trend to a reduced contraction amplitude by 20% (Figure 2D, right panel, p = 0.07 vs. WT) and toward a slower relaxation (Figure 2F, right panel) (time to 90% relaxation in s, CRE vs. WT, 0.70 ± 0.26 vs. 0.53 ± 0.19, p = 0.12 vs. WT) in line with the changes at the level of Ca^2+^ dynamics. Consequently, despite the absence of signs of ventricular hypertrophy, we detected changes in Ca^2+^ dynamics between isolated VCMs from CRE and WT mice.

### Changes in Ca^2+^ dynamics persist during β-adrenergic stimulation

**FIGURE 3:**
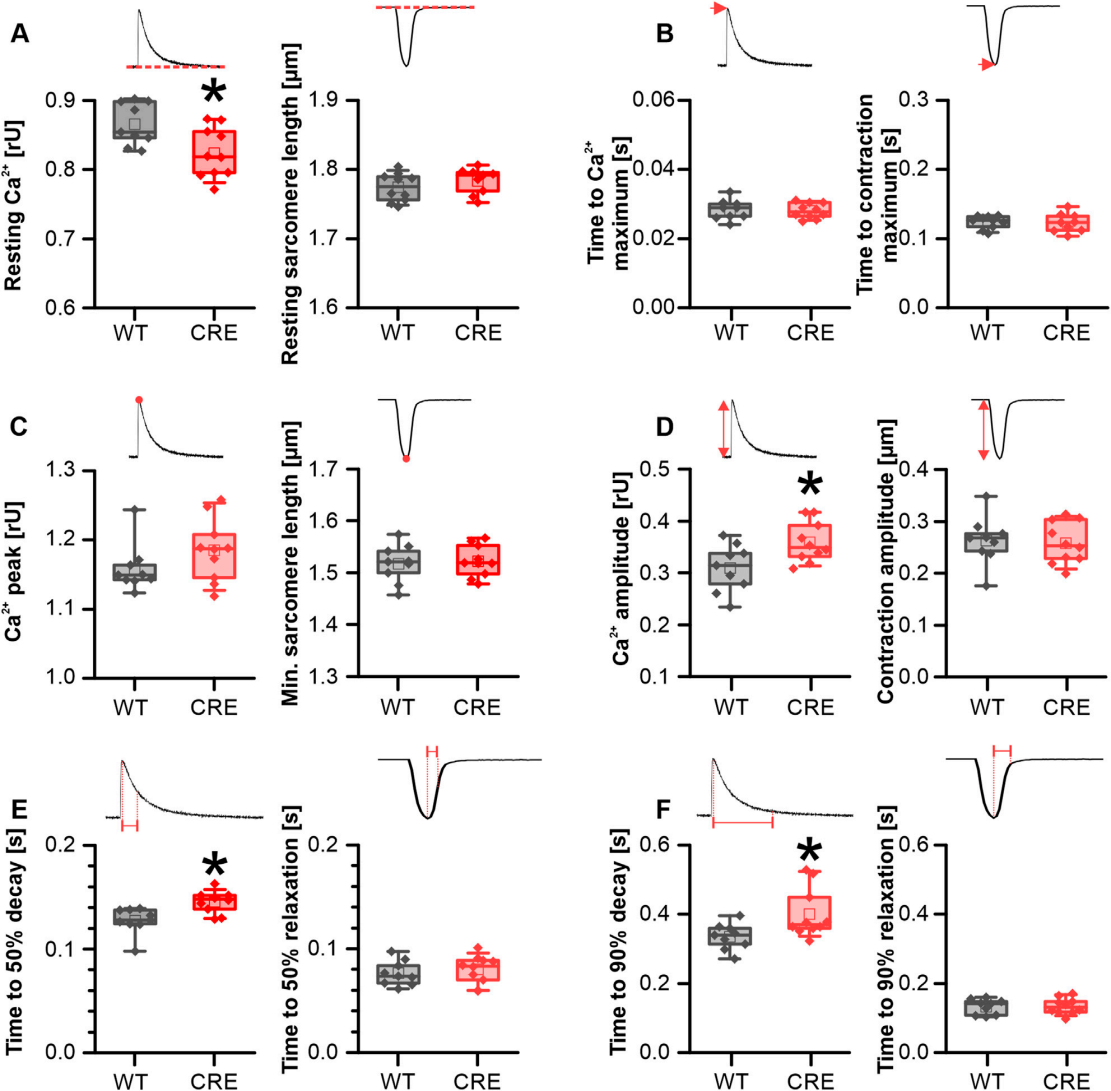
Alterations in Ca^2+^ dynamics persisted under β-adrenergic stimulation. Each panel shows the indicated parameter for Ca^2+^ transients (left) and sarcomere shortening (right) in VCMs from CRE and WT mice under acute stimulation with 10^–6^ M isoproterenol (ISO). Resting Ca^2+^ levels **(A)** remained reduced in VCMs from CRE vs. WT mice under ISO stimulation. Since peak Ca^2+^ levels **(C)** in CRE VCMs reached similar levels as in WT VCMs the Ca^2+^ transient amplitude **(D)** was increased in the CRE group. The delayed Ca^2+^ removal observed in CRE VCMs under basal conditions remained delayed vs. WT even under ISO stimulation as indicated by increased time to 50% **(E)** and 90% **(F)** decay of the Ca^2+^ transient. Again Ca^2+^ release speed **(B)** and all shortening parameters [**(A–F)**, right] were not different between groups (Number of Isolations, m/f, WT: n = 5/4; CRE: n = 5/5; each datapoint represents the median of 10 analyzed VCMs per isolation; *p < 0.05 vs. WT).

To analyze whether the observed changes under basal conditions were due to differential phosphorylation of proteins involved in Ca^2+^ dynamics between groups, we exposed VCMs acutely to the β-adrenergic receptor agonist isoproterenol (ISO; 1 µM). Also, under acute stimulation with ISO the resting Ca^2+^ level was reduced in VCMs from CRE versus WT mice by 4.9% (Figure 3A). Because peak Ca^2+^ reached comparable values under ISO in both groups (Figure 3C), the Ca^2+^ transient amplitude was even increased in ISO stimulated VCMs from CRE versus WT mice by 16% (Figure 3D). Again, the time to the maximum of the Ca^2+^ transient was not different between groups (Figure 3B). The slowed Ca^2+^ removal observed under basal conditions in the CRE group was still visible under acute ISO stimulation. The time required to reach 50% decay of Ca^2+^ (Figure 3E) was increased by 14% (p = 0.004 vs. WT) and the time to reach 90% decay of Ca^2+^ (Figure 3F) by 19.7% (p = 0.022 vs. WT) in VCMs from CRE versus WT mice. Consistently, τ was increased by 20% and the AUC of the decay phase by 43% (τ in s, CRE vs. WT, 0.18 ± 0.02 vs. 0.15 ± 0.01, p = 0.008 vs. WT; AUC in relative units*s, CRE vs. WT, 0.078 ± 0.016 vs. 0.054 ± 0.014, p = 0.004 vs. WT; data not shown). Again, the parameters of the sarcomere shortening were unaffected by the changes in Ca^2+^ dynamics, as shown in Figures 3A–F (right panels). In conclusion, changes at the level of Ca^2+^ dynamics in VCMs are preserved under acute β-adrenergic stimulation.

### Cre expression induces no significant changes in Ca^2+^ dynamics protein levels until 3 months

**FIGURE 4:**
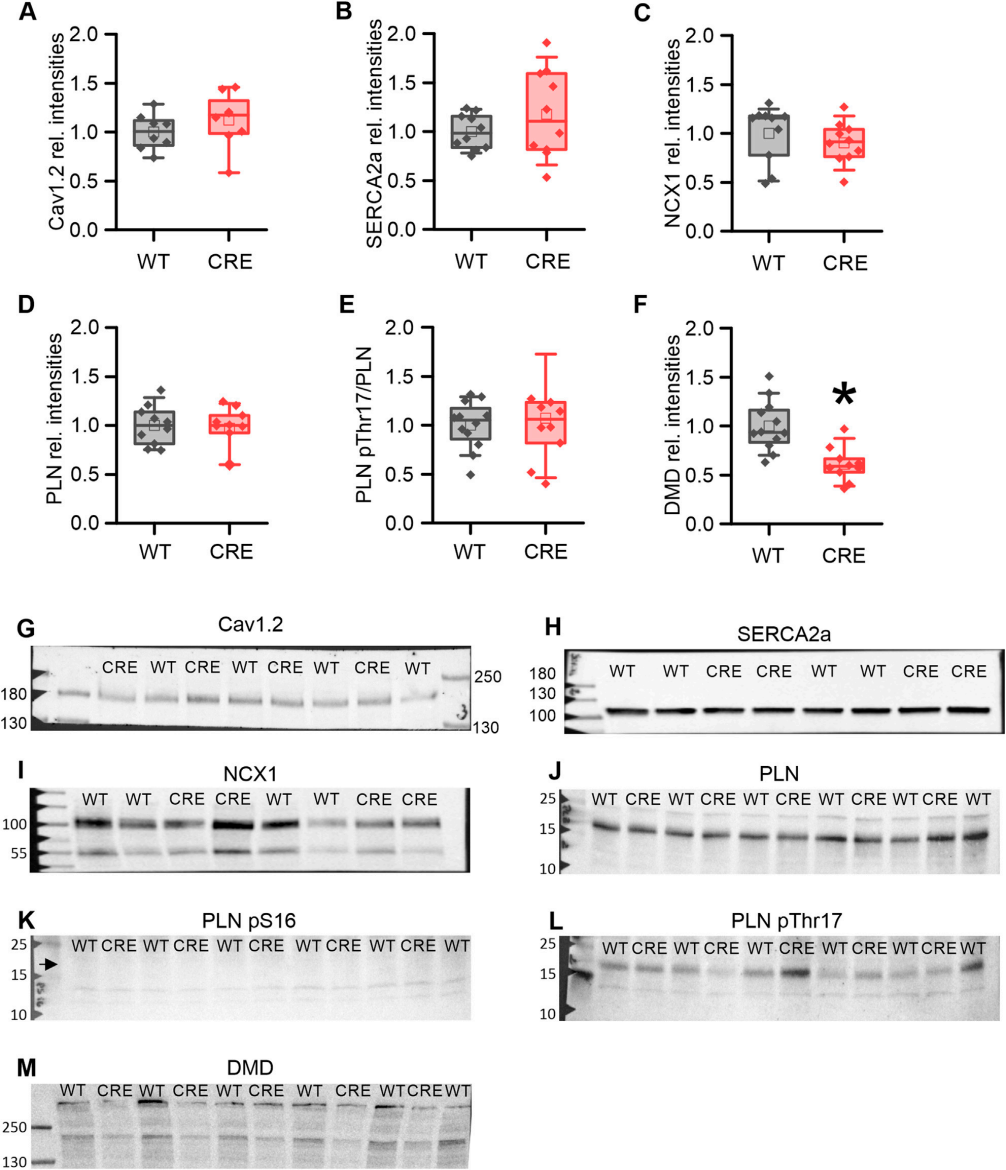
Protein levels of key Ca^2+^ regulators remain unchanged while dystrophin is reduced in the CRE group. Relative protein levels **(A–F)** and representative original Western blot images **(G–M)** of key proteins involved in Ca^2+^ dynamics and dystrophin as indicated. Band intensities were normalized to total protein (Ponceau) and then to the WT means. Protein levels of Cav1.2 **(A)**, SERCA2a **(B)**, NCX1 **(C)** total PLN **(D)**, and the relative PLN pThr17-phosphorylation **(E)** were not altered by Cre-expression. No specific signal for PLN pS16 phosphorylation **(K)** was detected (arrow), indicating no relevant PKA-dependent PLN phosphorylation under basal conditions. However, the level of dystrophin (DMD) was decreased in the CRE group **(F, M)** (n = 8-10 per group, equal samples per sex; *p < 0.05 vs. WT).

We proceeded to examine whether the changes in Ca^2+^ dynamics observed in the VCMs of 3-month-old CRE mice could be attributed to corresponding changes in the levels of Ca^2+^-regulating proteins. Protein levels of the pore-forming L-type Ca^2+^ channel subunit (Cav1.2) (Figures 4A, G), the cardiac sarcoplasmic/endoplasmic reticulum Ca^2+^-ATPase (Serca2a) (Figures 4B, H), the cardiac Na^+^-Ca^2+^-exchanger (NCX1) (Figures 4C, I), and total phospholamban (PLN) (Figures 4D, J) were not different between groups. In addition, we analyzed the phosphorylation of PLN at serine 16 (PLN pS16) and threonine 17 (PLN pThr17) in ventricular homogenates from shock-frozen hearts that had been perfused with Tyrode’s solution for 30 min in a Langendorff apparatus to allow for spontaneous beating and thus equilibration, a condition that should reflect the situation in isolated VCMs under basal conditions. We did not detect a relevant PLN S16-phosphorylation under basal conditions in either group (Figure 4K). The PLN Thr17-phosphorylation (Figures 4E, L) was unchanged between the two groups und thus not affected by Cre expression. Our results suggest that the changes in Ca^2+^ dynamics induced by Cre expression are not due to changes in the global levels of key regulatory proteins or their altered phosphorylation. However, Gillet et al. observed a reduction of dystrophin (DMD) at the mRNA and protein levels in αMHC-Cre mice and attributed the reduction of the L-type Ca^2+^ current I_Ca,L_ to this downregulation (Gillet et al., 2019). Consistent with the study of Gilet et al. dystrophin protein was almost halved in our study (Figures 4F, M; p = 0.001 vs. WT).

### Cre expression halves the mRNA for dystrophin

**FIGURE 5:**
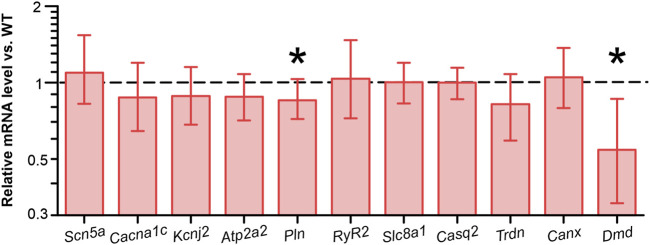
Reduced dystrophin and phospholamban mRNA levels in ventricular tissue from CRE mice Relative levels of mRNA encoding key Ca^2+^ regulatory proteins were assessed by real-time RT-PCR. The relative expression values and corresponding standard errors are displayed as calculated directly by REST software. While *Scn5a, Cacna1c, Kcnj2, Atp2a2, Ryr2, Slc8a1, Casq2, Trdn and Canx* levels were not significantly altered by Cre expression, *Pln* levels were significantly reduced compared to wild-type controls. mRNA levels of dystrophin (*Dmd*) were almost halved by Cre expression (n = 8 per group, equal samples per sex; *p < 0.05 vs. WT).

To identify potential transcriptional alterations in genes encoding pivotal proteins involved in Ca^2+^ dynamics, we conducted quantitative real-time RT-PCR on ventricular homogenates from CRE and WT mice. The levels of *Scn5a*, encoding the pore-forming subunit of the cardiac Na^+^ channel (Nav1.5), *Cacna1c*, encoding Cav1.2, *Atp2a2*, encoding Serca2a, *Slc8a1*, encoding NCX1, *Ryr2*, encoding the cardiac ryanodine receptor (RYR2), were not significantly different between groups (Figure 5). The levels of Casq2, Trdn and Canx, which encode the Ca^2+^-regulating cardiac proteins calsequestrin, triadin and calnexin, were also not significantly different between groups. However, we observed a slight but significant reduction in the relative *Pln* level in ventricular homogenates from CRE mice versus WT by 15% (p = 0.015 vs. WT). Given that the PLN protein functions as a brake for the SERCA2a-mediated Ca^2+^ transport into the sarcoplasmic reticulum, it may be postulated that this reduction represents an initial compensatory action against the retarded cytosolic Ca^2+^ removal observed in CRE mice. In addition, we examined the mRNA levels of Kcnj2, which encodes membrane potential stabilizing Kir2.1 channels, and found it to be unchanged between groups. The relative mRNA level of *Dmd* in CRE was almost halved compared to WT (relative mRNA level vs. WT with standard error, 0.543 [0.335–0.862], p = 0.002 vs. WT), confirming the reported finding.

## Discussion

The Cre/LoxP system is frequently used to generate mouse knockout models to investigate the function of individual target genes in cardiac tissue. This was made possible by the generation of a mouse model expressing the Cre recombinase under control of the αMHC (Myh6) promoter by the Schneider group more than 25 years ago (Agah et al., 1997). This model allows for the excision of floxed target genes exclusively in cardiomyocytes provided that Cre recombinase and the floxed target are simultaneously present in the same mouse. While additional models were developed that express Cre recombinase under various alternative promoters (Jiao et al., 2003; Parsons et al., 2004; Chen et al., 1998), the αMHC-Cre mouse remains the most prevalent model (Ronda and Roderick, 2024). In recent years, several studies have reported that Cre recombinase expression in cardiomyocytes is not inert, but in turn contributes to changes in cardiomyocytes that lead to age-dependent cardiotoxicity. The presence of degenerate loxP-like sites in the mouse genome has been discussed in the literature as the cause of this phenomenon (Pugach et al., 2015; Thyagarajan et al., 2000). However, Cre toxicity is not an exclusive property of the αMHC-Cre mouse. In αMHC-MerCreMer mice in which Cre is induced with tamoxifen, increased cardiomyocyte apoptosis, cardiac fibrosis, and both transient and chronic cardiac dysfunction were observed. (Bersell et al., 2013; Hall et al., 2011; Koitabashi et al., 2009).

The expression of an obvious phenotype in αMHC-Cre mice can be observed at the age of 6 months. At this age, authors observed reduced ventricular function (Pugach et al., 2015; Rehmani et al., 2019) dilated cardiomyopathy (Rehmani et al., 2019), fibrosis (Li et al., 2023; Pugach et al., 2015; Garbern et al., 2019), ventricular hypertrophy (Pugach et al., 2015; Li et al., 2023) and cardiomyocyte hypertrophy (Garbern et al., 2019). Li et al. reported that they systematically analyzed the cardiac function of αMHC-Cre mice with ECGs and concluded that there was no detectable phenotype in these mice from birth to 6 months of age (Li et al., 2023). Based on this earlier work, one might therefore be tempted to conclude that aMHC Cre mice do not show a significant phenotype until 6 months of age. However, a phenotype does not develop overnight, so it is not surprising that subtle effects of Cre recombinase expression can also be observed earlier. Pugach et al. observed a modest increase in relative *Anp* and *Bnp* mRNA level in αMHC-Cre mice versus wildtype controls at 3 months (Pugach et al., 2015). This was accompanied by slight increase in ejection fraction in male mice only. Anp and Bnp are early biomarkers for the diagnosis of heart failure but at the same time confer protective effects as antagonists of angiotensin II by relaxing vascular smooth muscle cells and increasing diuresis and natriuresis (Kerkelä et al., 2015). Also at 3 months of age, Gillet et al. reported a decrease in the L-Type Ca^2+^ current I_Ca,L_ in ventricular cardiomyocytes of αMHC-Cre mice (Gillet et al., 2019). Both findings may indicate a hemodynamic challenge in Cre mice and an associated impairment of Ca^2+^ dynamics.

In our study, we were indeed able to demonstrate an impairment of Ca^2+^ dynamics in VCMs of CRE vs. WT mice at an age of 3 months of age (Figures 2, 3), a time point at which we did not observe any signs of ventricular or atrial hypertrophy (Figure 1). Under basal conditions, where no β-adrenergic stimulation affects VCMs function, we observed a reduction in resting and peak Ca^2+^ levels and a delayed cytosolic Ca^2+^ transient decay. At the same time sarcomere shortening, which reflects VCM contractility, was not significantly affected (Figure 2). At 1 µM isoproterenol, where the maximal effect of β-adrenoceptor-mediated activation of Ca^2+^ dynamics by phosphorylation of PKA-dependent targets can be expected, the slower decay of the Ca^2+^ transient and the reduced resting Ca^2+^ were still visible (Figures 3A+E). Reduced resting cytosolic Ca^2+^ levels usually result from an altered balance between Ca^2+^ influx, storage and extrusion. In murine VCMs Ca^2+^ reuptake by SERCA into the sarcoplasmic reticulum (SR) is the predominant mechanism to lower cytosolic Ca^2+^ followed by Ca^2+^ extrusion via the sodium-calcium-exchanger (NCX) and the sarcolemmal Ca^2+^-ATPase (Schulte et al., 2016; Bers, 2008). However, we did not find significantly altered protein expression levels of SERCA or NCX1 when analyzing ventricular tissue from CRE and WT mice. Moreover, the Ca^2+^ transient decay was slower in CRE VCMs vs. WT, which does not indicate an increased Ca^2+^ extrusion rate at all. This slower decay of the Ca^2+^ transient was not dependent on the altered phosphorylation of SERCA-regulating PLN. Two observations support this conclusion. First, the basal phosphorylation levels of PLN at the PKA site S16 and the CaMKII site Thr17 were not different between groups (Figure 4). Dephosphorylated PLN inhibits SERCA2a by lowering its affinity for Ca^2+^. Phosphorylation of PLN at S16 or Thr17 increases Ca^2+^ affinity of SERCA2a leading to its disinhibition (Kranias and Hajjar, 2012). The phosphorylation state of phosphoproteins in cardiac tissue can be affected by the timing and exact procedure of tissue extraction. Therefore, in our study explanted hearts were allowed to beat spontaneously while perfused with Tyrode’s solution in a Langendorff apparatus for 30 min before shock freezing to achieve equilibrium conditions. Thus, the selected conditions for ventricular tissue preparation for the immunoblot analysis should reflect the basal measurement conditions of VCMs during Ca^2+^ transient recordings as close as possible. The second argument is that the slower decay of the Ca^2+^ transient was still visible under 1 µM isoproterenol (Figure 3E). At this concentration, at least PLN phosphorylation at S16 should be maximal and differences in phosphorylation that exist under basal conditions should disappear when total protein levels of SERCA2a and PLN are equal.

However, a possible explanation for the reduced resting cytosolic Ca^2+^ level is the reported decrease in I_Ca,L_ in αMHC-Cre VCMs (Gillet et al., 2019). A decrease in I_Ca,L_ reduces the amount of Ca^2+^ entering the cell, the amount of Ca^2+^ loaded into the SR and will also affect the amount of Ca^2+^ released from the SR. Sankaranarayanan et al. have shown that the inhibition of I_Ca,L_ by cadmium in rat ventricular cardiomyocytes reduced the diastolic Ca^2+^ level (Sankaranarayanan et al., 2017). Gillet et al. attributed the decrease in I_Ca,L_ to a reduction in dystrophin (DMD), which they had observed at the mRNA and protein level and in immunostaining of cardiomyocytes. We confirmed this finding and observed a significant reduction of *Dmd* mRNA and DMD protein levels compared to WT (Figures 4, 5). The actin-binding protein dystrophin plays a central role in the stability and function of cardiomyocytes. It links the cytoskeleton with the sarcolemma, ion channel proteins and other membrane proteins to the extracellular matrix (Dubinin and Belosludtsev, 2023). However, although dystrophin colocalizes with the L-type channel, the absence of dystrophin did not reduce I_Ca,L_ amplitude but did reduce I_Ca,L_ inactivation in dystrophin-deficient mdx mice (Sadeghi et al., 2002; Woolf et al., 2006; Koenig et al., 2014; Koenig et al., 2018). Prolonged inactivation of I_Ca,L_ would result in a prolonged Ca^2+^ influx into VCMs and most likely also affect CICR and Ca^2+^ transient decay, which is exactly what we observed in VCMs from αMHC-Cre vs. WT mice (Figures 2E, F). Since neither the protein levels of NCX, SERCA2a nor PLN and its phosphorylated forms were significantly changed between the groups (Figure 4), changes at the level of I_Ca,L_ are an obvious explanation for the delayed Ca^2+^ transient decrease we observed (Figures 2E, F). Moreover, Sadeghi et al. observed that the slowed inactivation of I_Ca,L_ in dystrophin-deficient mdx mice persisted under β-adrenergic stimulation (Sadeghi et al., 2002) which is in good agreement with our results since the slowed decay of the Ca^2+^ transient was still visible under β-adrenergic stimulation (Figure 3E). Obviously, β-adrenergic stimulation resulted in the same maximal cytosolic Ca^2+^ level in both groups (Figure 3C). Because resting Ca^2+^ was still lower in CRE VCMs (Figure 3A), the amplitude of the Ca^2+^ transient was increased in CRE VCMs compared with WT VCMs, probably indicating an increased fractional Ca^2+^ release from the SR. Again, the parameters of sarcomere shortening were not different between groups. One can speculate whether sarcomere shortening was not affected despite or because of the observed changes in Ca^2+^ dynamics since Cre-mediated changes on the level of the contractile apparatus cannot be ruled out but are beyond the scope of this study.

However, the main finding of our study is that the Ca^2+^ dynamics show clear changes, while this is not yet visible at the level of contractility or at the level of key proteins involved in Ca^2+^ dynamics. Focusing on the latter factors alone could mask the Cre-mediated changes already taking place and lead to the misinterpretation that no phenotype exists at the age of 3 months. Changes in Ca^2+^ can affect gene expression, a phenomenon called excitation-transcription coupling (Dewenter et al., 2017). Nuclear Ca^2+^ is influenced by cytosolic Ca^2+^. In addition, Ca^2+^-sensing or -dependent proteins such as calmodulin, calcineurin and the Ca^2+^/calmodulin-dependent kinase II (CaMKII) are activated by cytosolic Ca^2+^ oscillations and mediate transcriptional regulation by affecting the activity of transcription factors such as SRF, MEF2 and CREB1 (Dewenter et al., 2017; Blaeser et al., 2000; Sun et al., 1994). It is well documented that Ca^2+^/calmodulin-mediated activation of the phosphatase calcineurin leads to dephosphorylation of NFAT, nuclear translocation of NFAT and NFAT-mediated transcriptional activation of prohypertrophic genes, resulting in a pathological hypertrophic response (Molkentin, 2000; Molkentin et al., 1998; Dewenter et al., 2017). It is therefore possible that the observed early changes we have observed at the level of Ca^2+^ dynamics initiate the processes that ultimately lead to the changes documented later in Cre mice such as ventricular hypertrophy and reduced ventricular function.

### Limitations

The main aim of our study was to analyze whether αMHC-Cre expression mediates changes in Ca^2+^ dynamics at an early time point and not to identify possible underlying mechanisms in detail. Thus, our discussion remains somewhat speculative and detailed patch clamp analyses are needed to provide evidence for the proposed role of an altered I_Ca,L_ as responsible for the observed changes in the Ca^2+^ dynamics in CRE mice in our study. Total proteins levels, as measured in our study in homogenates by immunoblotting, do not indicate membrane localization or individual activity of these proteins, which may differ between the WT and CRE groups. Moreover, we cannot guarantee general validity of our findings for each αMHC-Cre mouse strain used in the scientific community. It is noticeable that the reports of the various working groups on the αMHC-Cre strain differ slightly with regard to the level of expression of the phenotype (Li et al., 2023; Pugach et al., 2015; Gillet et al., 2019; Rehmani et al., 2019; Garbern et al., 2019). It is discussed that the extent of cardiotoxicity might be related to the Cre expression level (Buerger et al., 2006; Bersell et al., 2013; Rashbrook et al., 2022). Buerger et al. generated Myh6-Cre mouse strains with different levels of Cre expression, compared them with an already established strain with low Myh6 dependent Cre expression (Abel et al., 1999), and reported that mice with low Cre expression were healthy, while mice with high expression developed heart failure (Buerger et al., 2006). Bersell et al. showed that the extend of cardiotoxicity in inducible Myh6-MerCreMer mice was dependent on the dose of tamoxifen which drives Cre activity (Bersell et al., 2013). Thus, all conditions that might influence the Cre expression level may affect the extent of cardiotoxicity. We used mice backcrossed to an FVB/N background - others used C57BL/6 (Pugach et al., 2015; Rehmani et al., 2019), which might influence the effects of Cre expression, as well as the housing conditions and the sex of the mice. Thus, each investigator must evaluate the impact of Cre expression in the mouse strain and under the respective conditions he or she uses.

## Conclusion

Finally, it can be concluded that processes that become visible in αMHC-Cre mice at 6 months of age begin much earlier. αMHC-Cre mice already show a measurable phenotype at 3 months of age that can influence essential cellular processes. It has already been pointed out by others that αMHC-Cre mice without the floxed target gene should serve as a control when investigating Cre-dependent knockout models. However, there are a considerable number of studies that do not use Cre controls as a control group (Rashbrook et al., 2022). Our study is intended to reiterate this call and to sensitize colleagues to subtle changes that may be initially overlooked.

## Data Availability

The raw data supporting the conclusions of this article will be made available by the authors, without undue reservation.
